# Frequency of suicide attempts and attitudes toward suicidal behaviour among doctors and nurses in Lagos, Nigeria

**DOI:** 10.4102/sajpsychiatry.v26i0.1402

**Published:** 2020-07-27

**Authors:** Olushola Olibamoyo, Olurotimi Coker, Abiodun Adewuya, Oluwaseun Ogunlesi, Olujimi Sodipo

**Affiliations:** 1 Department of Behavioural Medicine, Lagos State University College of Medicine, Lagos, Nigeria; 2 Department of Psychiatry, Lagos State University Teaching Hospital, Lagos, Nigeria; 3 Department of Family Medicine, Lagos State University Teaching Hospital, Lagos, Nigeria

**Keywords:** Attitudes, prevalence, suicidal behaviour, suicide attempt, doctors, nurses

## Abstract

**Background:**

Competence and attitudes toward suicidal behaviour affect practice. These attitudes may influence the consideration of suicide during personal crisis among doctors and nurses.

**Aim:**

The attitudes of doctors and nurses towards suicidal behaviour was assessed using the Attitudes Toward Suicide Scale (ATTS), which was validated in another study by the authors, evaluated for the possible factors affecting this relationship and estimated the frequency of suicide attempts among doctors and nurses.

**Setting:**

Lagos State University Teaching Hospital Lagos, Nigeria.

**Methods:**

The cross-sectional survey about attitudes toward suicide was done among 226 doctors and nurses working at a tertiary institute hospital in Lagos, Nigeria, using the ATTS. Sociodemographic profile and self-rated competence, commitment, empathy and irritation toward suicide were obtained. Stratified random sampling was used, data were analysed using Statistical Package for Sociological Sciences. Data was summarised, reliability of the ATTS was assured and variables compared by *t*-test and ANOVA. Independent predictors were identified via multiple regression (*p* ≤ 0.05).

**Results:**

Frequency of suicide attempts of 7.50% was found among respondents with a mean age of 35.84 ± 6.76 years. Attitudes toward suicidal behaviour were slightly positive (77.92 ± 9.90) and the independent predictors of less positive attitudes were nursing profession (β = 0.025, *p* < 0.001) and high self-rated irritation toward suicide (β = 0.18, *p* < 0.01).

**Conclusion:**

The frequency of suicide attempts is higher among doctors and nurses when compared to the general population. Doctors and nurses reported slightly positive attitudes toward suicidal behaviour with significant differences in the type of profession and levels of self-rated irritation toward suicide.

## Introduction

Suicidal behaviour can be conceptualised as a complex process that can range from suicidal ideation, which can be communicated through verbal or non-verbal means, to planning of suicide, attempting suicide and, in the worst case, suicide.^1^

Data on its epidemiology in Africa are limited due to lack of systematic collection of data in most countries with official statistics available in only 10% of low-income and middle-income countries^2^ which account for 85% of the world’s suicide, partly reflecting the population of those counties.^3^ Moreover, in some of these countries, for example Nigeria, attempted suicide is a criminal offence, leading to substantial under-reporting of non-fatal suicidal acts. Invariably, persons who engage in non-medically serious suicide attempts are unlikely to come to the authorities for help.^4^

Globally, 793 000 estimated suicide deaths occurred in 2016. In the same vein, a lifetime prevalence of suicide attempt stands at 2.70% worldwide.^5^ In fact, suicidal attempt may reach 20 times the number of completed suicide. This underscores the need for prevention of suicide in people with suicidal behaviour (ideation, plan and attempt).^6^

Competence and attitudes affect clinical practice and outcome of any intervention programme.^7^ This is particularly true for doctors and nurses who are a unique target for suicide prevention programmes, given their roles as gatekeepers with contact with high-risk patients.^8^ This fact holds regardless of their specialty. These attitudes may also influence the consideration of suicide at times of personal crisis among nurses and doctors, thereby explaining the increased risk of suicide in this group.^9^ To our knowledge, data on doctors’ and nurses’ suicide attempts are severely limited.

Measuring attitudes toward suicide is important for our understanding of the caregiving behaviour of the potential gatekeepers^10^ and in forming guidelines for decision-making in any intervention programme.^11^ Among the tools available for measuring attitudes toward suicide, the most representative include Suicide Opinion Questionnaire (SOQ), Suicide Attitudes Questionnaire (SUIATT) and Attitudes Toward Suicide Scale (ATTS)^12^.

We chose the ATTS for use in the study because it has fewer items (37 in number), is less complex and offers better reliability.^12^ In contrast, SOQ and SUIATT comprise 100 and 63 items. In addition, the ATTS has been used in a study done in two African countries (Ghana and Uganda) with similar cultures, thus allowing for comparison.^13^ Furthermore, it was validated by the same authors in this study although this validation is yet to be published.

Studies have shown that negative attitudes toward suicidal behaviour are associated with lack of preparation on suicide prevention among professionals, stigmatisation (and discrimination) and low level of care.^9^ Professional training on suicide to improve competence of medical professionals has been found to have a strong and positive association with favourable attitude and outcome.^14^

In Lagos and Nigeria at large, there is neither information nor data about the frequency of suicide attempts among doctors and nurses and their attitudes towards suicidal behaviour in spite of the fact that they are major stakeholders in any suicide prevention programme. Equally, possible factors affecting the relationship between their attitudes and sociodemographic factors, previous suicide attempts, self-rated competency, empathy, irritation and commitment towards suicidal behaviour remain unclear.

## Methods and materials

### Setting

This was a cross-sectional survey of doctors and nurses from the Lagos State University Teaching Hospital (LASUTH), Ikeja, Lagos, Nigeria. It serves about 20 million people with an average of 300 doctors and 350 nurses that attend to about 3000 patients daily (this figure was derived from the staff roll call and patient register in the emergency unit and clinics for 2017).

### Participants

Doctors and nurses employed full time by the hospital who returned the questionnaire.

### Instruments

A proforma questionnaire was developed by the authors based on the information required for the objectives of the study, target respondents, method of reaching the target respondents and length of the questionnaire. Preceding the development of the questionnaire, using a pilot study, information about sociodemographic profile, self-rated professional competence, commitment, empathy and irritation towards suicide behaviour were collected. The latter were scored on a three-point Likert scale (1 – low, 2 – average and 3 – high) based on responses from the pilot study.

The ATTS was developed from the SOQ by Renberg and Jacobsson.^15^ The instrument consists of three sections: firstly, contact with suicidal problems among significant others; the second section has 37 items touching on the individual’s opinion (attitudes) toward suicide on a five-point Likert scale that ranges from 1 (strongly disagree) to 5 (strongly agree). Based on this study, eight items (4, 5, 6, 12, 13, 29, 33 and 40) in the ATTS questionnaire were reversed scored, with lower scores indicating positive attitudes. The third section is about personal previous suicidal attempts. It has relatively high reliability and satisfactory face and construct validity.^12^ However, the internal consistency for the whole instrument and some of its factors was rather low.^15^ The ATTS has been validated in a study by the same authors following a pilot study which yielded a total Cronbach’s alpha of 0.72 to ascertain internal consistency following face and construct validity that were found satisfactory by two consultant psychiatrists, a family physician and a clinical psychologist. The main validation study yielded a 13-factor model which included 33 questions with an overall internal consistency of 0.68. The dimensions were found by factor analyses with varimax rotation and Kaiser normalisation. The factor model was generated based on eigenvalues, scree plot and factor loadings. The scores ranged from 33 to 165.

### Sample size

Three hundred (300) doctors and nurses working in the hospital were sent the paper survey in sealed envelopes.

### Procedure

The pool of participants was stratified in terms of profession (nurses and doctors) and specialty (family medicine, internal medicine, surgery, psychiatry, paediatrics, obstetrics and gynaecology, dentistry, emergency, ophthalmology, community health, ear, nose and throat, anaesthesiology and pathology) for equal distribution and spread from the roll call list made available by the hospital. Following stratification, the study participants were selected via random blocks of allocation from the stratified list by a senior academic not connected with the study, to give opportunity of everyone of being selected, leading to 150 doctors and 150 nurses spread along years of experience and specialty available in the hospital. Information from the survey was collected via paper questionnaires distributed in sealed envelopes with a letter of information in which the study was presented to all participants. This was done because of confidentiality and to allow participants to remain anonymous.

Participants were given two weeks to return the unaddressed sealed envelope to a box, reminders were sent to participants twice within the two weeks.

### Analysis

Data were coded, entered into the Statistical Package for Sociological Sciences version 23 and cleaned. Categorical variables were summarised with frequencies and percentages while continuous variables were summarised with their mean, mode, range and standard deviation.

Comparison of continuous data was done with student’s *t*-test and one way analysis of variance (ANOVA) between groups. Multiple regression analysis was used to identify independent predictors. Significance level was set at ≤ 0.05.

### Ethical consideration

Approval was obtained from the Ethics and Research Committee of Lagos State University Teaching Hospital, Ikeja, before the commencement of the study. Written informed consent was obtained from all participants after the aims and objectives of the study were explained to them via a letter of information. Confidentiality and voluntariness in participation were also explained.

## Results

Following two written reminders, 226 (75.33%) doctors and nurses returned the filled questionnaires to designated boxes; however, no doctor from the pathology specialty returned the questionnaire. The response rate differed slightly between doctors (111; 74.00%) and nurses (115; 76.70%). Their mean age was 35.84 ± 6.76 years and 151 (66.80%) were female; this proportion was similar to the sampled population of doctors and nurses.

Most (76.5%) reported that they had no training in suicidology and 17 (7.50%) participants reported having at least one suicide attempt during their lifetime. Among respondents with past suicide attempts, 11 (64.70%) were nurses, 13 (76.50%) were female and 11 (64.70%) were 30–39 years old. There were no significant associations between previous suicidal attempt and sociodemographic variables. The rest of the results are summarised in Table 1 and Table 2.

**TABLE 1:** Sociodemographic profile of the study population.

Variables	Doctors *n* = 111 (74.00%)		Nurses *n* = 115 (76.70%)		Total *n* = 226 (75.33%)	
	*n*	%	*n*	%	*n*	%
**Gender**						
Male	61	55.00	14	12.20	75	33.20
Female	50	45.00	101	87.80	151	66.80
**Age (years)**						
Range	25–50	-	21–58	-	21–58	-
Mean	35.86	-	35.81	-	35.84	-
Standard deviation	5.56	-	7.77	-	6.76	-
**Age group (years)**						
20–29	13	11.70	21	18.30	34	15.00
30–39	71	64.00	64	55.79	135	59.70
40–49	26	23.40	20	17.30	46	20.40
50–59	1	0.90	10	8.70	11	4.90
**Specialty**						
Family medicine	20	18.00	5	4.30	25	11.10
Internal medicine	10	9.00	20	17.40	30	13.30
Surgery	15	13.50	35	30.40	50	22.10
Psychiatry	6	5.40	4	3.50	10	4.40
Paediatrics	11	9.90	16	13.90	27	11.90
Obstetrics and gynaecology	7	6.30	11	9.60	18	8.00
Dentistry	13	11.70	6	5.20	19	8.40
Emergency	8	7.20	14	12.20	22	9.70
Ophthalmology	10	9.00	0	0.00	10	4.40
Community health	3	2.70	3	2.60	6	2.70
Ear, nose and throat	2	1.80	1	0.90	3	1.30
Anaesthesiology	6	5.40	0	0.00	6	2.70
**Years of practice**						
1–5	23	20.70	32	27.80	55	24.30
6–10	49	44.20	46	40.00	95	42.00
11–15	31	27.90	18	15.70	49	21.70
16–20	3	2.70	6	5.20	9	4.00
> 20	5	4.50	13	11.30	18	8.00
**Training in suicidology**						
Yes	16	14.40	37	32.20	53	23.50
No	95	85.60	78	67.80	173	76.50
**What degree of interest in suicidology training?**						
Not at all	10	9.00	7	6.10	17	7.50
A little degree	17	15.30	21	18.20	38	16.80
Some degree	58	52.30	40	34.80	98	43.40
Rather high degree	14	12.60	31	27.00	45	19.90
Very high degree	12	10.80	16	13.90	28	12.40
**Have you ever made any attempt to take your own life?**						
Yes	6	5.40	11	9.60	17	7.50
No	105	94.60	104	90.40	209	92.50

**TABLE 2:** Suicide attempt profile of the sampled population.

Variables	Have you ever made any attempt to take your own life?			
	Yes		No	
	*n*	%	*n*	%
**Occupation**				
Doctors	6	35.30	105	50.20
Nurses	11	64.70	104	49.80
**Gender**				
Male	4	23.50	71	34.00
Female	13	76.50	138	66.00
**Age group**				
20–29	1	5.90	33	15.80
30–39	11	64.70	124	59.30
40–49	4	23.50	42	20.10
50–59	1	5.90	10	4.80
**Specialty**				
Family medicine	4	23.50	21	10.00
Internal medicine	2	11.80	28	13.40
Surgery	3	17.60	47	22.50
Psychiatry	0	-	10	4.80
Paediatrics	2	11.80	25	12.00
Obstetrics and gynaecology	2	11.80	16	7.70
Dentistry	2	11.80	17	8.10
Emergency	1	5.90	21	10.00
Ophthalmology	1	5.90	9	4.30
Community health	0	-	6	2.90
Ear, nose and throat	0	-	3	1.40
Anaesthesiology	0	-	6	2.90
**Interest in suicidology training**				
Not at all	1	5.90	16	7.70
A little degree	3	17.60	35	16.70
some degree	5	29.40	93	44.50
rather high degree	4	23.50	41	19.60
very high degree	4	23.50	24	11.50
**Self-rated competence to manage a patient with suicidal behaviour**				
Low	12	70.60	83	39.70
Average	3	17.60	99	47.40
High	2	11.80	27	12.90

The mean score of the ATTS questionnaire among the participants was 77.92 ± 9.90 and it was normally distributed. On the independent *t*-test, nurses (*M* = 80.70, SD = 8.94) had significantly less positive attitudes toward suicide than doctors (*M* = 75.05, SD = 10.11), *t* (224) = -4.44, *p* < 0.001 (95% CI for mean difference -8.14 to -3.14). The results are presented in Table 3. Also, there was significant difference between female (*M* = 79.11, SD = 9.77) and male (*M* = 75.53, SD = 9.88) participants, *t* (224) = -2.58, *p* = 0.011 (95% CI for mean difference -6.33 to -0.83). Participants who had made previous attempts to take their own lives in the past had less positive attitudes overall (*M* = 80.47, SD = 12.42) than those who had not made an attempt to take their own lives (*M* = 77.71, SD = 9.70), *t* (224) = -1.10, *p* = 0.27; hence, previous suicidal attempts among doctors and nurses was not significantly associated with their attitudes toward suicidal behaviour in the study. However, there were significant differences regarding two of the subscales (factors) of the ATTS. When comparing respondents with at least one previous suicide attempt *(M* = 4.82, SD = 1.42) and those without previous suicide attempts *(M* = 5.47, SD = 1.18), *t* (224) =2.133, *p = 0.03* on believability of suicide ideation factor. Also, among the respondents with at least one previous suicide attempt *(M* = 6.59, SD = 2.76) and those without previous suicide attempts *(M* = 5.63, SD = 1.88), there was significant difference *t* (224) = -1.942, *p* = 0.05 on preventability of suicide factor. The results are presented in Table 4.

**TABLE 3:** Comparison of the validated 13-factor Attitudes Toward Suicide Scale scores among participants based on occupation (doctors and nurses).

Validated factors	Occupation				Test of significance
	Doctors		Nurses		
	Mean	SD	Mean	SD	
Ability to accept and understand suicide	12.58	3.91	5.46	1.08	*t* = −2.98**
Resignation	4.69	1.69	8.20	1.96	*t* = −2.78**
Preparedness to prevent suicide	3.37	1.40	3.64	1.46	*t* = −2.73**
Believability of suicide ideation	5.38	1.33	6.12	2.07	*t* = −0.51
Nature of suicidal attempts	6.87	1.89	4.95	1.89	*t* = −5.17***
Judgement about suicide	3.98	1.61	6.62	1.81	*t* = 1.65
Preventability of suicide	5.27	1.76	7.40	1.74	*t* = −3.33**
Communication and acceptance of assisted suicide	4.59	1.68	5.35	1.76	*t* = −1.49
Comprehensibility of suicide	6.97	1.85	6.00	1.33	*t* = 1.46
Norms	7.42	1.69	3.66	1	*t* = 0.07
Causality	5.07	1.62	80.7	8.94	*t* = −1.22
Loneliness and incomprehensibility	5.27	1.45	5.46	1.08	*t* = −3.88***
Unpredictability	3.57	1.01	8.20	1.96	*t* = −0.70
**Total**	**75.05**	**10.11**	**3.64**	**1.46**	t = −4.44***

* , *p* < 0.05;

** , *p* < 0.01:

*** , *p* < 0.001.

**TABLE 4:** Comparison of the validated Attitudes Toward Suicide Scale scores among participants based on participants who reported having at least one suicide attempt during their life time.

Validated ATTS factors	Previous suicide attempt				Test of significance
	YES		NO		
	Mean	SD	Mean	SD	
Ability to accept and understand suicide	14.71	4.86	13.24	3.81	*t* = −1.495
Resignation	5.06	1.64	4.99	1.65	*t* = −0.153
Preparedness to prevent suicide	3.82	1.47	3.62	1.47	*t* = −0.543
Believability of suicide ideation	4.82	1.42	5.47	1.18	*t* = 2.133*
Nature of suicidal attempts	7.24	1.56	7.57	2.07	*t* = 0.659
Judgement about suicide	3.64	1.37	3.82	1.56	*t* = 0.452
Preventability of suicide	6.59	2.76	5.63	1.88	*t* = −1.942*
Communication and acceptance of assisted suicide	5.24	1.44	4.74	1.82	*t* = −1.103
Comprehensibility of suicide	7.06	1.43	6.77	1.86	*t* = −0.623
Norms	7.71	2.26	7.39	1.66	*t* = −0.727
Causality	5.24	2.22	5.21	1.66	*t* = −0.057
Loneliness and incomprehensibility	5.82	1.42	5.63	1.44	*t* = −0.528
Unpredictability	3.52	1.23	3.62	0.99	*t* = 0.364
**Total**	**80.47**	**12.43**	**77.72**	**9.7**	**t = −1.100**

* , *p* < 0.05;

** , *p* < 0.01:

*** , *p* < 0.001.

Self-rated competence, commitment, empathy and irritation in regard to attending to patients with suicidal behaviour was analysed using ANOVA as shown in Tables 5–7. Attitudes toward suicide was significantly different between levels of self-rated competence, *F* (2223) = 4.13, *p* = 0.017. Post-hoc analyses using Tukey’s test indicated those who perceived their competence to be high (*M* = 81.41, SD = 10.62) had significantly less positive attitudes toward suicide compared to the average self-rated group (*M* = 76.05, SD = 9.33), *p* = 0.027. Also there was significant difference between levels of self-rated sense of irritation towards patients with suicidal behaviour, *F* (2223) = 6.43, *p* = 0.002. Post-hoc analyses using Tukey’s test showed that participants that rated their sense of irritation either high (*M* = 82.06, SD = 8.89) or average (*M* = 80.95, SD = 8.79) had significantly less positive attitudes toward suicide than the low group (*M* = 76.32, SD = 10.09), *p* = 0.048 and *p* = 0.007. There were no significant differences according to levels of empathy and commitment toward patients with suicidal behaviour.

**TABLE 5:** Attitudes toward suicidal behaviour measured with the validated Attitudes Toward Suicide Scale according to self-rated competence, commitment, empathy and irritation toward patients’ suicidal behaviour.

Variables	High		Average		Low	
	M	SD	M	SD	M	SD
Competence	81.41	10.62	76.06	9.33	78.86	10.01
Irritation	82.06	8.89	80.95	8.76	76.32	10.09
Empathy	77.08	10.40	79.27	9.10	79.80	8.30
Commitment	77.61	9.89	77.07	10.14	80.95	9.08

**TABLE 6:** One-way analysis of variance of validated Attitudes Toward Suicide Scale scores in levels of perceived competence.

Source	Degree of freedom	Sum of squares	Mean of squares	*F*	*p*
Between groups	2	791.82	395.91	4.13	0.017*
Within groups	223	21385.90	95.90	-	-
Total	225	22177.72	-	-	-

* , *p* < 0.05.

**TABLE 7:** One-way analysis of variance of Attitudes Toward Suicide Scale scores in levels of irritation.

Source	Degree of freedom	Sum of squares	Mean of squares	*F*	*p*
Between groups	2	1208.73	604.37	6.43	0.002**
Within groups	223	20968.99	94.03	-	-
Total	225	22177.72	-	-	-

** , *p* < 0.01.

Multiple regression analysis was carried out to investigate whether occupation (doctor or nurse), gender, self-rated competence in management of suicidal behaviour and irritation toward suicidal behaviour in patients significantly predicted positive attitudes toward suicidal behaviour. The result of the regression indicated that the four predictors produced *R*^2^ = 0.118, *F* (4221) = 7.43, *p* < 0.001. It was found that occupation significantly predicted positive attitudes toward suicidal behaviour *(β* = 0.25, *p* < 0.001*),* as did self-rated irritation *(β* = 0.18, *p* < 0.01). The rest of the results are summarised in Table 8

**TABLE 8:** Multiple regression analysis for variables predicting attitudes toward suicidal behaviour as measured by the validated Attitudes Toward Suicide Scale.

Variable	Unstandardised beta	Standard error	Standardised beta
Occupation	4.85	1.47	0.25***
Gender	1.06	1.49	0.05
Self-rated competence	−0.001	0.95	0.07
Self-rated irritation	2.84	1.01	0.18**
*R*^2^	11.80	-	-
*F*	7.43**	-	-

* , *p* < 0.05;

** , *p* < 0.01;

*** , *p* < 0.001.

## Discussion

The main findings were lifetime frequency of suicide attempts among doctors and nurses of 7.50%, attitude toward suicidal behaviour as measured by the ATTS questionnaire was slightly positive (77.92 ± 9.93), there were significant differences in attitudes in terms of occupation (doctors and nurses), gender, perceived self-rated competence and irritation toward suicidal behaviour. The independent predictors were occupation and self-rated irritation. There are no published studies on attitudes toward suicidal behaviour in Nigeria; hence direct comparison of findings might be difficult.

All over the world, there is a paucity of data on suicide attempts among health workers. Where data on suicidal attempts is available, it is among the general population and the quality is low. This explains the overall under-reporting of suicide attempts. In this study, frequency of suicide attempts among doctors and nurses was 7.50%. This represents a sharp increase when compared with other lifetime prevalence of suicide attempt in the general population such as the WHO estimates of 2.70%,^5^ 0.40 – 4.20% found in the WHO multisite intervention study on suicidal behaviour community survey of low-income and middle-income countries^16^ and 0.70% found by Gureje et al.^17^ in the Nigerian survey of mental health and well-being.

This suggests that suicidal attempts are more common among doctors and nurses than in the general population and the same effect has been observed in completed suicides among doctors and nurses.^18,19^ Also, our findings are consistent with observations on suicide attempts in the general population that show a higher prevalence in women.^20,21^ Doctors and nurses in our study with previous suicide attempts predominantly belonged to the 30–39 years age group which was close to what was observed in a similar study^20^ which reported a mean age of 38.52 ± 8.52 years among physicians and similar to that observed for completed suicide in physicians.^22^ Suicide attempts was higher among nurses than doctors which is consistent with another study.^23^

The plausible explanations for the higher frequency compared to the general population may be a reluctance to seek help during periods of personal crisis due to stigma or fear of career implications even when they are convinced they need it.^23,24^ Rightfully, they fear lack of confidentiality when receiving mental health care as private conversations with therapists could be turned over to medical boards and illegally accessed by their supervisors at their institutions.

This is further complicated by the ‘physician heal thyself’ culture promoted by society and the public perception that maintains that doctors are successful, intelligent, wealthy and immune from the problems of the masses. To patients, it is inconceivable that doctors and nurses could have the highest suicide rate of any profession. The stress and nature of their work schedule and personality traits such as perfectionism and competitiveness can make them vulnerable. Moreover, they have access to means of harming themselves.^18,19,23,24,25,26^

In addition, according to Nigeria’s penal code, chapter 27, section 327, ‘any person who attempts to kill himself is guilty of a misdemeanour, and is liable to imprisonment for one year’; Nigerian law also criminalises abetment of suicide.^4^ According to chapter 27, section 326 of the Nigeria penal code:

Any person who (1) procures another to kill himself; or (2) counsels another to kill himself and thereby induces him to do so; or (3) aids another in killing himself; is guilty of a felony, and is liable, to imprisonment for life.^4^

Therefore, it can be argued that data about rates of suicide attempts are likely to be highly unreliable and possibly under-reported due to fear of prosecution in the general population, unlike the doctors and nurses in this study who anonymously filled the questionnaires, with the assurance that the data would not be used for any other purpose than research. This could be a possible reason of the wide margin between the 0.70% from the Nigerian survey and 7.50% from this study.

The association between previous suicide attempts and attitudes toward suicidal behaviour among respondents was not significant. However, this study showed that respondents with a history of at least one previous attempt to die by suicide had less positive attitudes towards suicide when compared to respondents with no previous suicidal attempts. This result is in line with previous studies.^27^

The findings at the subscale (factor) levels of the ATTS showed that respondents with previous suicidal attempts had more positive attitudes toward the believability of suicidal ideation factor when compared to respondents with no previous history of suicide attempts. This association was significant. This factor included items, such as ‘risk to evoke suicidal thought if asked about it’, ‘suicide is considered for a long time’ and ‘people who make threats seldom complete suicide’. Also, respondents with previous history of suicide attempts showed less positive attitudes toward preventability of suicide factor when compared to respondents with no history of previous suicidal attempts and the association was significant. This factor included items such as ‘suicide can be prevented’, ‘give help to commit suicide if severe, incurable disease people’ and ‘suicide should not always be prevented’.

For the non-significant association between suicidal attempt and attitudes of the respondents toward suicide, it can be argued that suicidal behaviour is due to complex interplay of so many factors, of which attitude is just one. Since other factors known to significantly influence suicidal behaviour such as access to means, psychiatric illnesses, unemployment and substance abuse were not incorporated into the study,^27^ a further study that will include these elements should be considered in the future.

Furthermore, the pattern of less positive attitudes toward suicide among respondents with a history of previous suicide attempts could be as a result of the attitude being a coping strategy of denial, suppression and self-accusation in a cultural and professional context, where it is not always possible to verbalise and communicate suicidal problems.^27^ Regarding the significant associations seen at the subscale (factor) level, a plausible reason could be that suicidal ideation tends to influence a non-condemning attitude towards suicidal behaviour while suicidal attempts tend to influence condemning attitudes toward suicidal behaviour due to cultural taboo associated with it. The different patterns presented put forward several interesting angles that will require further investigation within the context of suicide prevention. However, it is beyond the scope of the study.

For the multiple regression analysis, our *R*^2^ was low (11.80%). However, it was entirely expected especially when trying to predict human behaviour; furthermore, we had statistically significant predictors which allowed us to draw important conclusions.

Nurses had significantly less positive attitudes to suicidal behaviour when compared to doctors both on correlation and regression analysis and this is in line with some other studies done in different countries;^28,29^ the greatest differences between the professions in this study were seen in the attitudes ‘Ability to accept and understand suicide’, ‘Resignation about suicide’, ‘Preparedness to prevent suicide’, ‘Nature of suicidal attempts’, ‘Preventability of suicide’ and ‘Loneliness and incomprehensibility’.

Explanations are possible for these differences: it may be due to differences in training between doctors and nurses in Nigeria. For example, a doctor requires a medical degree and a nurse requires a nursing certificate or diploma most of the time. Also, the doctor’s training includes a compulsory psychiatry posting unlike the training of most nurses. In other words, for nurses, mental health training is only an area of specialisation after qualification. Lastly, the difference may be as a result of continued education among the doctors sampled (most of them undergoing residency training).

Although there is no evidence that these differences have any clinical impact on suicide prevention among study participants, it is noteworthy that further training, workshops and continued medical education on suicide prevention can help to improve knowledge and attitudes toward suicidal behaviour and can affect care decisions.^30^ Also, attitudes of doctors and nurses toward suicide may influence not only their motivation to treat patient in suicidal crisis or during deliberate self-harm behaviour but also their ability to address the patients’ problems^30^. As a consequence, this will further increase the risk of suicide in patients.^30^

Furthermore, in the African context, doctors and nurses are key opinion leaders in their community, and in most social and religious settings are in a power category whose attitudes can inform the views held by the community.^31^ Possibly influencing prevalence of suicidal attempts and completed suicide. This is why one of the recommended ways of preventing suicide has been increase in awareness among health care workers of their own attitudes and taboos towards suicide and its prevention.^31^

The female gender was found to have significantly less positive attitudes toward suicidal behaviour on correlation. This was incongruent with previous studies.^10,32^ However, on further analysis, using multiple regression to identify independent predictors, the difference was not significant. The possible explanation is that there were more women (66.80%) in the study and the majority (87.80%) of the nurses were female in the study.

Lastly, self-rated irritation towards suicidal behaviour was significantly associated with less positive attitudes toward suicidal behaviour. This is supported by Grimholt et al.^33^ who suggested that positive attitudes include empathy, compassion and acceptance of shortening someone’s life full of suffering. The relationship is inversely proportional to positive attitudes toward suicidal behaviour. A plausible reason may be bidirectional negative attitudes may make them judgmental and make them irritated at those with suicidal behaviour or their irritation towards suicidal behaviour may hinder them from learning or partaking in activities (training, workshops, etc.) that may positively influence their attitudes toward patients with suicidal behaviour.

There are a few limitations that should be considered when interpreting the findings. The data were self-reported and not based on observed behaviour, making it impossible to eliminate social desirability effect. The same respondents were used for the validation and correlation and the findings may not be totally representative of the doctors and nurses in Lagos, Nigeria, particularly those not working in a tertiary hospital such as where this study took place.

Despite these limitations, it is very important to note that this is the first study from Nigeria to look at the frequency of suicide attempts among doctors and nurses. This distinct study used a validated ATTS to systematically inquire about the attitudes toward suicide with stratified random sampling method and the respondents were sampled across different specialties, age groups and levels of seniority, in order to ensure heterogeneity and representation of the population studied. Lastly, the response rate of 75.33% was also significant thereby minimising response bias.

## Conclusion

The lifetime frequency of suicide attempts is higher among doctors and nurses compared to the general population. Doctors and nurses reported slightly positive attitudes towards suicidal behaviour with significant differences in type of profession and levels of self-rated irritation towards suicide. The differences could be as a result of level of education and training required to be qualified to practise these professions, therefore a need for continuous training on suicidology is required.
